# PReS-FINAL-2262: Arthritis in a patient with type 1 glycogen storage disease

**DOI:** 10.1186/1546-0096-11-S2-P252

**Published:** 2013-12-05

**Authors:** E Baskin, US Bayrakci, H Sakallı, A Kantarcı, F Ozcay

**Affiliations:** 1 Pediatric Nephrology and Rheumatology, Baskent University, Ankara, Turkey; 2 Pediatric Gastroenterology, Baskent University, Ankara, Turkey

## Introduction

Glycogen storage diseases, the commonest of which is Von Gierke disease, are characterized by excessive deposit of glycogen in liver and muscle owing to the lack of key enzymes in glycogen metabolism.

## Objectives

The occurrence of gout in patients with Von Gierke disease may be related to acidosis causing a decrease in uric acid excretion by the proximal tubule and routing of glucose-6-phosphate to the pentose pathway.

## Results

A 7 year-old girl was admitted to out patient clinic with a swollen and painful toe associated with lethargy and poor appetite; there was no rash, serositis, or eye inflammation, no muscle pain or weakness. Her history revealed that she had been followed up with a diagnosis of glycogen storage disease type 1 and neutropenia since she was 1 year-old. She had recurrent attacks of pain and edema in her toes, ankles and hip which lasts for a while and resolved sometimes by itself sometimes by non-steroidal anti-inflammatory drugs. Physical examination revealed marked edema, induration and pain in the first toe of right foot as well as hepatomegaly.

Serum investigations revealed iron deficiency, neutropenia, elevated erythrocyte sedimentation rate, c-reactive protein and fibrinogen levels. Serum uric acid, lactate, and fasting triglycerides were elevated as well. MEFV mutation analysis was negative. She was given colchicines for her gout, allopurinol for high serum uric acid levels and diet counseling to help regulate his blood glucose through the day. She was followed up for 4 years with colchicines with only few attacks of gouty arthritis.

## Conclusion

Colchicine could be the drug of choice in the treatment of gouty arthritis with life style management and appropriate diet. Though liver transplantation would be the definitive treatment for glycogen storage disease, its effect on gouty arthritis is still controversial.

## Disclosure of interest

None declared.

